# 

**Published:** 1896-08

**Authors:** 


ESTABLISHED 1855. SUBSCRIPTION, $2.00.

### ATLANTA MEDICAL AND SURGICAL JOURNAL.

VOLUME XIII. NEW SERIES. ATLANTA, GA., AUGUST, 1896. No. 6.

